# Impact of Rothman index on delay of ICU transfer for hematology and oncology patients deteriorating in wards

**DOI:** 10.1186/s13054-018-2268-6

**Published:** 2018-12-04

**Authors:** Neal Fitzpatrick, Daniel Guck, Andry Van de Louw

**Affiliations:** Division of Pulmonary and Critical Care Medicine, Penn State Health Hershey Medical Center, 500 University Dr, Hershey, PA 17033 USA

**Fig. 1:**
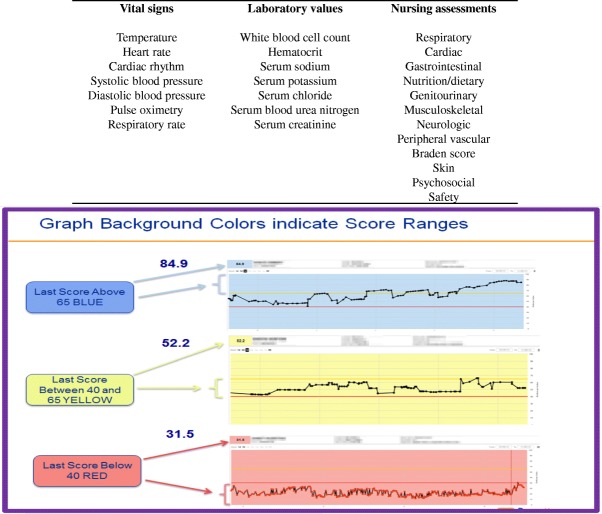
Table presenting clinical, laboratory and nursing components used to compute the Rothman index. Graph displaying examples of Rothman index with color codes associated with risk of deterioration, as may appear on monitors in nursing stations. Each of three windows represents a different patient and displays course of Rothman index over time. Horizontal yellow and red lines represent thresholds used to define patients at medium risk and high risk of deterioration respectively

Delayed ICU admission is associated with increased mortality in patients with malignancies [1]. Early warning scores have been proposed to prevent delay in ICU transfer but their impact on outcome remains uncertain [2]. Recently, the Rothman index (RI), a more comprehensive score collecting 26 variables (Fig. 1), has been shown to predict 24-h and hospital mortality [3, 4], performing better than the Modified Early Warning Score [4]. The RI is indexed from 100 and reduced to a minimum of − 91 as a function of increasing risk. We assessed whether implementation of the RI at our institution decreased the delay of ICU transfer or the severity of illness on ICU admission for hematology/oncology patients deteriorating on wards.

We performed a before/after study comparing 86 patients transferred from wards to the ICU before RI implementation (the RI was computed from electronic medical records but not available to staff) with 86 consecutive patients transferred after RI implementation and staff training. We collected the lowest RI within 24 h prior to ICU transfer (low RI), the delay between low RI and ICU transfer, and whether and when patients reached validated alarms of 40 (high risk) and 20 (very high risk) for the RI prior to transfer. The SOFA score on ICU admission, vital organ support in the ICU and mortality were collected.

Post-RI patients were older and had higher Charlson comorbidity index (Table 1). The two periods included similar proportions of patients with hematological malignancy and bone marrow transplant (50% and 15% respectively). None of the severity indexes (cardiac arrest at day 1, mechanical ventilation or vasopressor requirements within 24 h, lactates and SOFA score on ICU admission) was different between the two groups. Similarly, none of the RI-derived indexes evaluated (low RI, delay in low RI–ICU transfer, proportion of patients reaching high-risk or very high-risk alerts and delay between these alerts and ICU transfer) differed between pre-RI and post-RI patients. About 75% and 40% of patients reached high-risk and very high-risk RI alerts prior to ICU transfer. The ICU and hospital mortality were 36% and 46% respectively for the whole population.

**Table 1:** Main characteristics and comparison of hematology and oncology patients transferred to the ICU from the ward before (pre-RI) and after (post-RI) implementation of the Rothman index (RI)

	Pre-RI (*n* = 86)	Post-RI (*n* = 86)	*p*
Age (years)	60 (49–67)	65 (60–73)	0.0003
Gender, male/female, (n)	44/42	53/33	0.19
Charlson comorbidity index	5.0 (3.0–7.0)	6.0 (4.3–8.8)	0.0005
Hematological malignancy, n(%)	52 (60)	46 (53)	0.36
HSCT, n(%)	14 (16)	17 (20)	0.55
Lactates (mmol/L)	1.7 (1.1–3.0)	1.8 (1.2–3.1)	0.65
Cardiac arrest day 1, n(%)	7 (8)	5 (6)	0.55
Sepsis day 1, n(%)	52 (60)	56 (65)	0.53
Mechanical ventilation day 1, n(%)	25 (29)	29 (34)	0.51
Duration of mechanical ventilation (days)	1 (0–3.8)	0 (0–2)	0.08
Vasopressors day 1, n(%)	29 (34)	32 (37)	0.63
RRT, n(%)	19 (22)	21 (24)	0.72
SOFA score day 1	7.0 (6.0–10.8)	7.0 (5.0–10.0)	0.68
ICU mortality, n(%)	26 (30)	36 (42)	0.11
Hospital mortality, n(%)	36 (42)	43 (50)	0.25
Lowest RI	25.5 (12.8–39.4)	22.7 (12.5–39.3)	0.61
Delay lowest RI—ICU (h)	2.0 (0.3–10)	2.0 (0.1–5.0)	0.25
Alert RI < 40, n(%)	67 (78)	64 (74)	0.59
Alert RI < 20, n(%)	32 (37)	37 (43)	0.40
Delay RI < 40—ICU (h)	14 (2–24)	15 (5–24)	0.63
Delay RI < 20—ICU (h)	9 (1–22)	5 (2–13)	0.39

Results presented as median (interquartile range) for continuous variables and number (percentage) for categorical variables. Pre-RI and post-RI patients compared using the Wilcoxon rank-sum test and Fisher’s exact test for continuous and categorical variables respectively

*ICU* intensive care unit, *HSCT* hematological stem cell transplant, *RRT* renal replacement therapy, *SOFA* Sequential Organ Failure Assessment

In this small population of oncology patients deteriorating in wards, implementation of the RI did not result in patients being transferred to the ICU earlier or with fewer organ failures. This raises concerns about staff training and proper use of the RI in routine. More studies are warranted to translate this sophisticated and expensive tool into survival benefits for the patients.
